# Effectiveness of Smartphone-Based Community Case Management on the Urgent Referral, Reconsultation, and Hospitalization of Children Aged Under 5 Years in Malawi: Cluster-Randomized, Stepped-Wedge Trial

**DOI:** 10.2196/25777

**Published:** 2021-10-20

**Authors:** Griphin Baxter Chirambo, Matthew Thompson, Victoria Hardy, Nicole Ide, Phillip H Hwang, Kanika Dharmayat, Nikolaos Mastellos, Ciara Heavin, Yvonne O'Connor, Adamson S Muula, Bo Andersson, Sven Carlsson, Tammy Tran, Jenny Chen-Ling Hsieh, Hsin-Yi Lee, Annette Fitzpatrick, Tsung-Shu Joseph Wu, John O'Donoghue

**Affiliations:** 1 Faculty of Health Sciences Mzuzu University Mzuzu Malawi; 2 Malawi eHealth Research Center University College Cork Cork Ireland; 3 University of Washington Seatle, WA United States; 4 Imperial College London London United Kingdom; 5 University College Cork Cork Ireland; 6 College of Medicine University of Malawi Blantyre Malawi; 7 Lund University Lund Sweden; 8 Luke International Norway Mzuzu Malawi

**Keywords:** community case management, mobile health, pediatrics, childhood infection, mobile phone

## Abstract

**Background:**

Integrated community case management (CCM) has led to reductions in child mortality in Malawi resulting from illnesses such as malaria, pneumonia, and diarrhea. However, adherence to CCM guidelines is often poor, potentially leading to inappropriate clinical decisions and poor outcomes. We determined the impact of an e-CCM app on the referral, reconsultation, and hospitalization rates of children presenting to village clinics in Malawi.

**Objective:**

We determined the impact of an electronic version of a smartphone-based CCM (e-CCM) app on the referral, reconsultation, and hospitalization rates of children presenting to village clinics in Malawi.

**Methods:**

We used a stepped-wedge, cluster-randomized trial to compare paper-based CCM (control) with and without the use of an e-CCM app on smartphones from November 2016 to February 2017. A total of 102 village clinics from 2 districts in northern Malawi were assigned to 1 of 6 clusters, which were randomized on the sequencing of the crossover from the control phase to the intervention phase as well as the duration of exposure in each phase. Children aged ≥2 months to <5 years who presented with acute illness were enrolled consecutively by health surveillance assistants. The primary outcome of urgent referrals to higher-level facilities was evaluated by using multilevel mixed effects models. A logistic regression model with the random effects of the cluster and the fixed effects for each step was fitted. The adjustment for potential confounders included baseline factors, such as patient age, sex, and the geographical location of the village clinics. Calendar time was adjusted for in the analysis.

**Results:**

A total of 6965 children were recruited—49.11% (3421/6965) in the control phase and 50.88% (3544/6965) in the intervention phase. After adjusting for calendar time, children in the intervention phase were more likely to be urgently referred to a higher-level health facility than children in the control phase (odds ratio [OR] 2.02, 95% CI 1.27-3.23; *P*=.003). Overall, children in the intervention arm had lower odds of attending a repeat health surveillance assistant consultation (OR 0.45, 95% CI 0.34-0.59; *P*<.001) or being admitted to a hospital (OR 0.75, 95% CI 0.62-0.90; *P*=.002), but after adjusting for time, these differences were not significant (*P*=.07 for consultation; *P*=.30 for hospital admission).

**Conclusions:**

The addition of e-CCM decision support by using smartphones led to a greater proportion of children being referred to higher-level facilities, with no apparent increase in hospital admissions or repeat consultations in village clinics. Our findings provide support for the implementation of e-CCM tools in Malawi and other low- and middle-income countries with a need for ongoing assessments of effectiveness and integration with national digital health strategies.

**Trial Registration:**

ClinicalTrials.gov NCT02763345; https://clinicaltrials.gov/ct2/show/NCT02763345

## Introduction

Malawi has one of the world’s lowest doctor-to-patient ratios [1], with ≤1 doctor per 50,000 people [2-5], which is considerably lower than the World Health Organization’s recommendation of 1 doctor per 5000 people. In order to improve access to essential health services for many of Malawi’s marginalized communities, the Malawi Ministry of Health started an Emergency Human Resources Program in 2005 when it recruited and trained selected community members to become health surveillance assistants (HSAs) [6]. HSAs are allocated to hard-to-reach areas, each serving a population of approximately 1000 [7]. They have several duties they are expected to carry out (eg, family planning, environmental health, and HIV counseling), of which community case management (CCM) is integral [8]. HSAs are encumbered by an overwhelming workload, insufficient day-to-day professional support (including training opportunities), and inadequate remuneration [7]. These competing pressures may lead to further progress in disease control and childhood survival. CCM is a paper-based clinical decision aid that enables frontline community health workers known as HSAs in Malawi to recognize and manage the major causes of mortality among children aged ≤5 years in low- and middle-income countries (LMICs), specifically malaria, pneumonia, diarrhea, and malnutrition [9]. CCM uses simple signs and symptoms to help HSAs identify self-limiting conditions that can be managed in the community and more serious illnesses that require medical attention from clinically trained health personnel (eg, nurses) at higher-level health care facilities or hospitals. The introduction of this strategy has provided access to basic health care that many children would otherwise have been denied [10].

While mortality rates among the under-fives have steadily declined since the introduction of CCM in 2009, concerns have begun to surface about inconsistent standards of delivery of CCM; it has been observed that HSAs do not always follow all the steps in the protocol when assessing children, potentially resulting in inappropriate clinical management decisions [11]. In Malawi, HSAs deliver CCM from village clinics; in a country where 84% of the population resides in rural locations [12] more than 8 km from facility-based health providers, CCM has helped provide equitable access to health care for many communities that were previously underserved [13]. CCM is widely implemented in LMICs and has demonstrated some success in improving the management of children with acute illness and reducing mortality. For example, the CCM of pneumonia reduced pneumonia-associated mortality by 70% in some developing countries [14,15]. As the adoption of CCM in Malawi in 2008, this strategy has contributed to significant reductions in under-five mortality from 97.8 per 1000 live births to 52.7 in 2017 [16].

The implementation of CCM is based on the diagnostic and prognostic value of certain signs and symptoms; children with uncomplicated illness are managed in the community with symptomatic treatment, whereas those with *danger* signs are urgently referred to a health facility that can provide a higher level of care. Accurate and timely urgent referral is an integral component of CCM and is a prerequisite for improving health outcomes among acutely unwell children. Aside from preventable mortality, failure to refer at the appropriate time can overburden constrained resources at higher-level facilities needed for treatment of severely unwell children, lead to unnecessary hospital admissions, or involve caregivers repeating inconvenient journeys to village clinics because their child’s condition has not resolved or is deteriorating.

However, there is considerable evidence of suboptimal management decisions from field studies, including poor HSA adherence to the required steps of CCM [17]. HSAs do not always follow all the steps in the protocol when assessing children, potentially resulting in inappropriate clinical management decisions [11]. Indeed, because CCM is delivered using a paper-based tool, it is easy for relevant fields to be overlooked or for clinically invalid data to be recorded, contributing to inappropriate management decisions. Poor adherence to CCM is further compounded by factors such as transportation costs or distance to higher-level facilities or medication supply chain issues that further impede the potential impact of CCM.

The proliferation of mobile phones and cellular networks in LMICs has prompted the development of several mobile health (mHealth) technologies as alternative platforms for delivering childhood intervention strategies [18]. Decision support systems within these technologies can facilitate clinical decision-making and offer potential advantages over paper-based tools [19,20]. The potential advantages of e-CCM over paper-based CCM include shorter consultation time and a greater likelihood of correct diagnostic and treatment decisions, as noted in previous studies [21,22]. However, adopting mHealth tools as an adjunct (or replacement) for current paper-based assessment and reporting tools requires considerable initial and sustained investment. Therefore, demonstrating the impact of mHealth on health care outcomes is crucial. This study aims to determine the effectiveness of smartphone-based CCM on urgent referral, reconsultation, and hospitalization of children aged ≤5 years in Malawi**.**

## Methods

### Study Design

The full study protocol has been published [23]. In summary, we used a stepped-wedge, cluster-randomized design trial to compare paper-based CCM with a mobile phone app version of the CCM that was developed by the Supporting LIFE research team (SL e-CCM app) [24]. Village clinics were grouped into 6 clusters based on geographic proximity, and clusters were randomized to determine the sequence of crossover from the control (using paper CCM alone) to the intervention (using paper CCM as well as SL e-CCM) and the duration of exposure (2-7 weeks) in each phase. Children aged ≥2 months to <5 years were triaged using paper CCM or paper CCM+SL e-CCM app depending on when they presented to village clinics.

This study was conducted in 102 village clinics across Nkhata Bay and Rumphi districts in northern Malawi, where Chichewa, Chitumbuka, and Tonga are the principal languages spoken. Cassava farming is the main occupation of the 215,429 inhabitants of the Nkhata Bay District, situated on Lake Malawi. Rumphi district, which extends west from Lake Malawi to the Zambian border, is principally a tobacco farming community with 166,460 inhabitants. According to the National Statistic Office of Malawi, 23.4% and 17.4% of the adult population (defined as those aged ≥15 years) are illiterate in Nkhata Bay and Rumphi, respectively. Village clinics are typically basic community structures equipped only with rudimentary diagnostic aids (eg, stopwatch) and medical supplies (eg, oral rehydration therapy and artemisin-based combination therapy). Each clinic is operated by a single HSA, who is a government-employed community health worker responsible for assessing and managing acutely unwell children using CCM. HSAs are people who possess a Malawi School Certificate of Education or Junior Certificate of Education. They are frontline health workers who work in the village clinic [7]. A village clinic serves a catchment area of approximately 1000 people [10,25]. Both districts are predominantly rural, with 30%-35% of the population residing more than 8 km from a health facility with qualified clinical staff [26].

### Participants

A total of 102 HSAs were recruited to participate (men: 77/102, 75.4%; women: 24/102, 23.5%; not disclosed: 1/102, 0.9%). The ages of these HSAs ranged from 27 to 59 years, and they had been working as HSAs for between 1 and 27 years (mean 10.1 years). Before the trial, 101 HSAs already had phones, of which 36.6% (37/101) were smartphones.

HSAs consecutively enrolled eligible children aged ≥2 months to <5 years from November 2016 to February 2017, who presented with acute illness but were not unconscious, convulsing, or previously enrolled in the study. Because of high rates of illiteracy in Nkhata Bay and Rumphi districts, caregivers were required to verbally consent in their preferred language for their child to be enrolled to avoid limiting participation by literacy levels.

### Randomization and Blinding

Village clinics were grouped into 6 clusters based on geographic proximity. Clusters were then randomized using a web-based random number generator to determine the sequencing of crossover of clusters from the control phase (paper CCM) to the intervention phase (paper CCM+SL e-CCM), as well as the duration of exposure (2-7 weeks) in each phase. Neither the participants nor the researchers were blinded to allocation. Baseline data were collected during the index visit and recorded in the village clinic register (VCR) in the control arm and in the SL e-CCM app during the intervention phase.

### HSA Training

Before commencing recruitment, we engaged with national and district-level health authorities to explain to them the objectives of the clinical trial and the procedures to be done. HSAs recruited by the study team attended a 1-day training workshop to learn study procedures for the control and intervention phases of the trial, as well as how to operate the SL e-CCM app and the smartphone (details of the hardware and development and functionality of the app are described in full elsewhere [23]). Before crossover from the control to the intervention phase, HSAs attended a further 2-day training workshop to familiarize them with study procedures and the technology specific to the intervention phase. Training workshops were conducted in English with a member of the research team fluent in the regional dialects of Chichewa, Tonga, and Chitumbuka to facilitate communication. HSAs were permitted to use their clinical discretion if they disagreed with the care recommendations provided by the SL e-CCM app.

### Procedures

During the control phase, HSAs used paper-based CCM to assess and manage children for 2-7 weeks (depending on the assigned order of clusters after randomization). Details of clinical presentations were recorded by hand in the VCR, as per standard practice. During the intervention phase, HSAs recorded clinical presentations synchronously in both the VCR and SL e-CCM apps. Form validation ensured that HSAs were required to complete every field on the e-CCM app [11]. A complete inventory of the data collected by HSAs during each phase of the trial has been reported elsewhere [23]. Outcome data were collected retrospectively using local nursing students or graduates who traveled to village clinics and higher-level health facilities within an assigned catchment area on a weekly basis to manually abstract data from patient records*.* Enrolled children were reidentified at clinical sites by cross-referencing each child’s full name, date of enrollment, date of birth, and sex along with caregiver name and contact details prepopulated on clinical research forms. Given that caregivers can represent to any village clinic and higher-level health facility, child identifiers and subsequent attendances at health facilities were verified by caregivers via cell phone beforehand, to improve the efficiency of data collection. Clinical data were recorded in the VCR during both the control and intervention phases of the study in order to fulfill local reporting requirements for village clinics and district health authorities.

### Outcomes

The primary outcomes were urgent referrals to higher-level health facilities, reconsultations to village clinics, and hospitalization within 7 days of study enrollment.

Outcomes were determined by case note review of VCR and health facility records undertaken 2-weeks after study enrollment. Reconsultations were defined as reattendances to any village clinic for a health concern related to the reason for presentation recorded in the VCR at baseline, and hospital admissions related to an inpatient stay in a secondary or tertiary care facility for ≥1 day because of deterioration of illness recorded in the VCR.

### Description of SL e-CCM App

**Figure 1:**
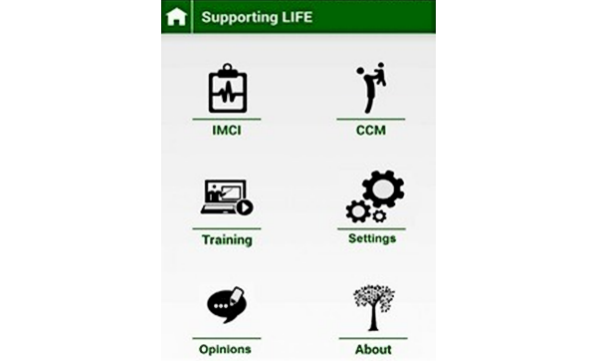
The Supporting LIFE App. CCM: community case management; IMCI: Integrated Management of Childhood Illnesses.

**Figure 2:**
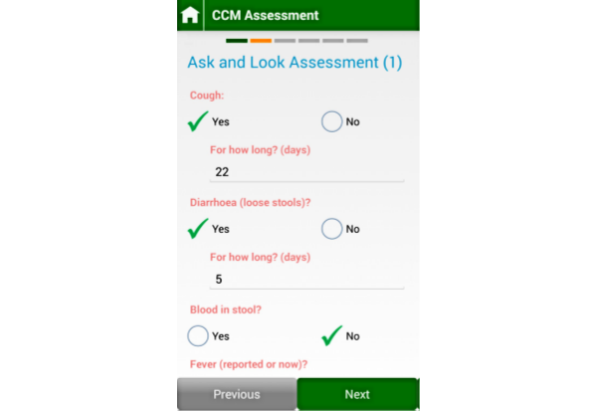
CCM assessment using the Supporting LIFE App. CCM: community case management.

The SL e-CCM App was developed and implemented by the Supporting LIFE research team and is an android-based smartphone app that replicates the paper-based CCM decision support tool routinely used by HSAs in Malawi and across sub-Saharan Africa [27]. The tool enables HSAs to enter the information they would usually gather using the paper CCM form, answer a series of clinical questions, and obtain selected clinical measurements (eg, breathing rate). Data are entered directly into the app via touch screen technology, either by selecting the appropriate option or by entering free text [23,28]. The app provides treatment recommendations, including medications, referral to health facilities, and health education for parents. The app requires that users complete all steps (ie, questions and prompts) before the user can proceed to the next step. Figures 1 and 2 show the dashboard of the Supporting LIFE App.

### Statistical Analysis

Descriptive analyses were used to examine the individual-level baseline characteristics of children between the control and intervention periods across all clusters. Participant characteristics included the child’s age, sex, the district in which the child was recruited, and the treatment decision by the HSA at the index visit. For continuous measures, we reported the mean and SD. For categorical measures, we reported counts and proportions. The 2-tailed *t* tests and chi-square tests were used to determine bivariate differences for continuous and categorical measures, respectively.

Following an intention-to-treat principle, we evaluated overall differences in the proportion of children who were urgently referred to a higher-level health facility by HSAs between the control and intervention periods. For the primary analysis, urgent referrals to higher-level facilities were evaluated at the patient level using multilevel mixed effects models [29]. As all outcomes were binary, a logistic regression model with random effect of cluster and fixed effect for each step was fitted [30,31]. Potential confounders included baseline factors of age, sex, and geographical location of village clinics (ie, urban and rural). Calendar time was also adjusted for in the analysis, and the effect of time was modeled as a categorical variable [30]. Odds ratios (ORs) with 95% CI were estimated and reported. Potential effect modification by age and sex was assessed by stratification and inclusion of interaction terms with the intervention period in the model. Other main outcomes of reconsultations at village clinics and hospitalizations were also analyzed using mixed models with the same potential confounders and effect modifiers. For secondary analyses, we explored the heterogeneity in treatment effects between clusters using within-cluster comparisons between the intervention and control periods. Statistical significance was set at *P*<.05, and all tests were two-sided. All analyses were conducted using Stata version 14 (StataCorp). Patient flow, including the number and reasons for exclusion and withdrawal, was reported according to CONSORT recommendations.

### Dissemination of Results

The results of this study will be disseminated through presentations in research conferences, and copies of this study will be put in the Libraries of Mzuzu University, College of Medicine, University of Washington, University College Cork, Lund University, and Imperial College London.

## Results

### Participants Recruited and Baseline Characteristics

A total of 6965 children were recruited, of whom 49.11% (3421/6965) were included in the paper CCM (control) phase and 50.88% (3544/6965) were included in the e-CCM (intervention) phase (Table 1). A larger number of children were recruited from the Nkhata Bay district than from Rumphi in both trial phases, reflecting the higher population in the former, as noted above. Children recruited in the control phase were significantly younger (mean age 24.6, SD 15.4 months) than those in the intervention phase (26.9, SD 16.1 months). Most children in both the control (3217/3421, 94.03%) and in the intervention phases (3335/3544, 94.10%) were treated at home after their assessment by the HSA (Table 1).

**Table 1:** Baseline characteristics of participants (N=6965).

Characteristic			Control arm (paper-CCM^a^; n=3421)		Intervention arm (e-CCM app+paper CCM; n=3544)		*P* value
Age (months), mean (SD)			24.6 (15.4)		26.9 (16.1)		<.001
**Sex, n (%)**							
	Male	1741 (50.89)		1783 (50.31)		.63	
	Female	1680 (49.10)		1759 (49.63)		.66	
	Not recorded	0 (0)		2 (0.06)		.16	
**District^b^, n (%)**							
	Rumphi	1590 (46.47)		1535 (43.31)		.01	
	Nkhata Bay	1777 (51.94)		1962 (55.36)		<.001	
**HSA^c^ treatment decision at index visit, n (%)**							
	Urgent referral	179 (5.23)		198 (5.58)		.51	
	Treated at home or advice given to caregiver	3217 (94.03)		3335 (94.10)		.91	
	Nothing recorded in village clinic register	25 (0.73)		11 (0.31)		.01	

^a^CCM: community case management.

^b^Information on districts was missing for 54 participants in the control group (1.6%) and 47 participants in the intervention group (1.3%).

^c^HSA: health surveillance assistant.

### Effect of Intervention on Urgent Referral

A total of 5.23% (179/3421) of children in the control phase were urgently referred to a higher-level health care facility, compared with 5.58% (198/3544) in the intervention phase (OR 1.04, 95% CI 0.83-1.29; *P*=.75; Table 2). After adjusting for the effects of time (model 1), the OR was 2.02 (95% CI 1.27-3.23; *P*=.003), in favor of the intervention. Further adjustment for age, sex, and district (models 3 and 4) provided similar strengths of association. We found no significant effect modification in the tested models. Examination of cluster-specific treatment effects, adjusted for calendar time, across the 6 clusters showed that the direction of effect was identical to the overall effect (ie, favoring intervention) in 4 of the 6 clusters but was statistically significant in only one of these (cluster 2; *P*<.001) most likely affected by sample size. In 2 of the 6 clusters (clusters 3 and 4), the direction of effect favored the control arm but was statistically significant in only one of these clusters (cluster 4; *P*=.009).

**Table 2:** Effects of intervention on urgent referral (N=6965).

Model			Control arm (n=3421), n (%)		Intervention arm (n=3544), n (%)		OR^a^ (95% CI)		*P* value		ICC^b^ (95% CI)
**Urgent referral**			179 (5.23)		198 (5.58)						
	Model 1: unadjusted	N/A^c^		N/A		1.04 (0.83-1.29)		.75		0.128 (0.042-0.326)	
	Model 2: adjusted for time	N/A		N/A		2.02 (1.27-3.23)		.003		0.127 (0.042-0.325)	
	Model 3: adjusted for time, age, and sex	N/A		N/A		2.01 (1.26-3.21)		.003		0.125 (0.041-0.321)	
	Model 4: adjusted for time, age, sex, and district	N/A		N/A		2.15 (1.44-3.22)		<.001		0.024 (0.000-0.054)	

^a^OR: odds ratio.

^b^ICC: intracluster correlation coefficient.

^c^N/A: not applicable.

### Effect of Intervention on Repeat Consultation

Repeat consultations with an HSA within 7 days occurred for 5.26% (180/3421) of children in the control phase, compared with 2.42% (86/3544) in the intervention phase (OR 0.45, 95% CI 0.34-0.59; *P*<.001; Table 3). After adjusting for the effects of time (model 1), the OR was 0.57 (95% CI 0.32-1.04), in favor of the intervention arm, but this was not statistically significant (*P*=.07). Further adjustment for age, sex, and district (models 3 and 4) provided similar ORs. We found no significant effect modification in these models. Examination of cluster-specific treatment effects showed that the direction of effect for the outcome of repeat consultation favored the control arm in 4 clusters (clusters 1, 3, 4, and 5) and was statistically significant in only one of these (cluster 4; *P*=.04), whereas in clusters 2 and 6 it favored the intervention, but was not statistically significant (cluster 2, *P*=.79; cluster 6, *P*=.09).

A total of 219 clinical features were recorded on the children attending repeat consultations, although not all children had clinical symptoms recorded, and more than one clinical feature could be recorded in one child. The most common symptoms reported were fever for <7 days, cough for 21 or more days, diarrhea for <14 days without blood, and fast breathing (Table 3).

**Table 3:** Effects of intervention on repeat consultation (N=6965).

Model		Control arm (n=3421), n (%)	Intervention arm (n=3544), n (%)	OR^a^ (95% CI)	*P* value	ICC^b^ (95% CI)
**Repeat consultation**		180 (5.3)	86 (2.4)			
	Model 3: adjusted for time, age, and sex	N/A^c^	N/A	0.57 (0.32-1.04)	.07	0.186 (0.063-0.435)
	Model 4: adjusted for time, age, sex, and district	N/A	N/A	0.58 (0.32-1.05)	.07	0.084 (0.024-0.250)

^a^OR: odds ratio.

^b^ICC: intracluster correlation coefficient.

^c^N/A: not applicable.

### Effect of Intervention on Hospital Admission

A total of 565 children were admitted to the hospital, 9.35% (320/3421) in the control arm and 6.91% (245/3544) in the intervention arm (OR 0.75, 95% CI 0.62-0.90; *P*=.002; Table 4). Of these, 51.3% (290/565) were urgently referred by their HSA, and 47.8% (270/565) were taken to the hospital by their parents (information on origination was not available for 5 children). After adjusting for the effects of time (model 2), the OR was 1.23 (95% CI 0.83-1.81), in favor of the control arm, but was not statistically significant (*P*=.30). Further adjustment for age, sex, and district (models 3 and 4) provided similar ORs. We found no significant effect modification in these models. Examination of cluster-specific treatment effects showed that the direction of effect on hospital admission favored the control arm in 4 clusters (clusters 1, 2, 5, and 6) and was statistically significant in only one of these (cluster 2; *P*=.002), and the intervention arm in 2 clusters (3 and 4), but was not statistically significant (cluster 3, *P*=.49; cluster 4, *P*=.10).

**Table 4:** Effect of intervention on hospital admissions (N=6965).

Model			Control arm (n=3421), n (%)		Intervention arm (n=3544), n (%)		OR^a^ (95% CI)		*P* value		ICC^b^ (95% CI)
**Hospitalization**			320 (9.4)		245 (6.9)						
	Model 3: adjusted for time, age, and sex	N/A^c^		N/A		1.23 (0.83-1.82)		.29		0.034 (0.010-0.113)	
	Model 4: adjusted for time, age, sex, and district	N/A		N/A		1.14 (0.78-1.67)		.50		0.004 (0.000-0.036)	

^a^OR: odds ratio.

^b^ICC: intracluster correlation coefficient.

^c^N/A: not applicable.

## Discussion

### Principal Findings

We found that the addition of e-CCM to the usual practice of assessing and treating children with HSAs using the paper-based assessment tool led to a significant increase in the proportion of children referred urgently to higher-level health care facilities. This direction of effect of the intervention was found in most but not all of the 6 clusters of clinics. The intervention was also associated with smaller proportions of children who attended a repeat consultation at the village clinic or who needed to be admitted to the hospital, but these were not statistically significant after full adjustment in the models.

We speculate that the modest effect of e-CCM on increasing the proportion of children who were urgently referred may have been because of greater adherence to the CCM decision support algorithm [32,33], as the smartphone app encouraged adherence to CCM [33], or that the smartphone app reduced errors by providing more accurate assessment than paper CCM alone. This is because when using e-CCM, there is less opportunity for the HSA to miss assessment items, as they are forced to complete all items before progressing in the assessment tool. As such, this reduces errors that may have occurred when HSAs use paper-based CCM alone, where they can choose to fill in some questions and leave out others. The effects could, however, merely represent the impact of the requirement in our trial for HSAs to double enter data (ie, on paper format and then using the app) with replication of assessment providing more opportunity to conduct the required assessment. Children in the intervention group were less likely to return for repeat consultation, which could be because of their assessment being more thorough or correct at their initial visit to the village clinic, thus reducing the need to return to the clinic for the same illness.

Our trial also demonstrated that 94.1% (6552/6965) of children aged ≤5 years presenting with acute illness are managed in the community by HSAs, with only approximately 1 in 20 being urgently referred to a higher-level health care facility. Some caregivers appear to bypass the village clinic and attend the hospital directly without being assessed at the village clinic. This implies dissatisfaction with the services provided by lower-level health care facilities, such as village clinics [25]. One reason that caregivers may bypass village clinics and go directly to higher-level facilities may be because caregivers perceive that their child needs urgent attention and may feel that going to the village clinic wastes time [34]. Indeed, previous studies have found that some community members do not have confidence in the care they receive from first-level health care providers, such as community health workers [35,36].

A major strength of our study is the use of a rigorous stepped-wedge design, which aims to minimize potential biases. Previous research has established considerable support by HSAs and the local community for planned interventions [7,10,37]. We could not conduct an individual randomized study at the child or HSA level, following guidance from local investigators. A stepped-wedge design has significant advantages over a simple before and after design as it attempts to minimize potential temporal biases and provides a more powerful study design using a control group. We selected referrals as the main trial outcome as this is a major driver of health care use, and there is currently evidence that underreferral contributes to child morbidity [38,39]. We believe that this study provides a high degree of generalizability to similar settings in sub-Saharan Africa, where CCM is used.

This study had some limitations. One involved the requirement of our study design for HSAs to continue to use the paper tool during the intervention phase, which meant that we could not assess the independent impact of e-CCM but rather its benefit. This occurred because the SL e-CCM App was not yet endorsed by the Malawi Ministry of Health, so it could not replace the paper tool. In addition, assessing outcomes was challenging because of the lack of unique patient identifiers and incomplete records at health care facilities. The study may have been underpowered to detect effects on repeat consultations and hospital admission, and we noted heterogeneity between clusters in direction and significance of effects.

Our results add to previous qualitative studies that have shown that HSAs feel empowered using apps such as the one evaluated here [21] and that these tools might support greater adherence to CCM [32]. These results support wider implementation and potential addition of further functionalities to support HSAs using smartphones to facilitate other tasks, such as reporting requirements or drug stocking. For policy makers, our study provides robust evidence on the effectiveness of this e-CCM tool that few other studies have provided to date and could therefore add evidence to support national digital health strategies in developing an integrated community health information system. However, we acknowledge that further data are needed to determine the costs (and cost-effectiveness) of the initial setup (training, smartphones, and servers), and ongoing support (technical support, hardware replacement, and software updates). For researchers in this area, evidence for the independent effects of e-CCM on outcomes is needed. These results call for additional studies on e-CCM apps and similar mHealth tools to determine the impact on other clinical outcomes, such as duration of illness and resource use. In addition, there is a need to ensure data and workflow integration of e-CCM as part of community health service delivery, management, and existing digital tools used at other levels of the health system, such as the District Health Information System, electronic medical record systems, and real-time surveillance at the community level.

### Conclusions

The vast majority of children assessed by HSAs in these 2 large rural areas of Malawi are managed by HSAs in their communities. Adding an e-CCM that involved an app on smartphones to HSAs’ usual paper-based CCM tool led to a significant increase in the proportion of children referred from village clinics to higher-level health care facilities. Although the effects on decreased hospital admission and decreased repeat consultation were suggestive but inconclusive, they support the hypothesis that the e-CCM tool improved decision-making at the HSA level. Combined with existing qualitative literature showing high levels of acceptability of mHealth versions of CCM by community health workers, our findings support further efforts to deploy smartphone-based tools as part of integrated digital health strategies in Malawi and similar countries in sub-Saharan Africa, with ongoing evaluation of effectiveness, cost-effectiveness, and acceptability by health care workers and community members.

## Abbreviations

CCM: community case management
HSA: health surveillance assistant
LMIC: low- and middle-income country
mHealth: mobile health
OR: odds ratio
SL e-CCM: Supporting LIFE e-community case management
VCR: village clinic register
